# Liquid crystals of neat boron nitride nanotubes and their assembly into ordered macroscopic materials

**DOI:** 10.1038/s41467-022-30378-5

**Published:** 2022-06-07

**Authors:** Cedric J. Simonsen Ginestra, Cecilia Martínez-Jiménez, Asia Matatyaho Ya’akobi, Oliver S. Dewey, Ashleigh D. Smith McWilliams, Robert J. Headrick, Jesus A. Acapulco, Lyndsey R. Scammell, Michael W. Smith, Dmitry V. Kosynkin, Daniel M. Marincel, Cheol Park, Sang-Hyon Chu, Yeshayahu Talmon, Angel A. Martí, Matteo Pasquali

**Affiliations:** 1 Department of Chemical and Biomolecular Engineering, Rice University, 6100 Main Street, MS 369, Houston, TX 77005 USA; 2 Department of Chemistry, Rice University, 6100 Main Street, MS 369, Houston, TX 77005 USA; 3 Department of Chemical Engineering and The Russell Berrie Nanotechnology Institute (RBNI), Technion-Israel Institute of Technology, 3200003 Haifa, Israel; 4 BNNT Materials, LLC, 300 Ed Wright Lane Suite A, Newport News, VA 23606 USA; 5 Department of Physics and Optical Engineering, Rose-Hulman Institute of Technology, 5500 Wabash Ave, CM 169, Terre Haute, IN 47803 USA; 6 Advanced Materials and Processing Branch, NASA Langley Research Center, Hampton, VA 23681 USA; 7 National Institute of Aerospace, 100 Exploration Way, Hampton, VA 23666 USA; 8 Department of Materials Science and NanoEngineering, 6100 Main Street, MS 369, Houston, TX 77005 USA; 9 Department of BioEngineering, 6100 Main Street, MS 369, Houston, TX 77005 USA; 10 The Smalley-Curl Institute, Rice University, 6100 Main Street, MS 369, Houston, TX 77005 USA

**Keywords:** Molecular self-assembly, Mechanical properties, Synthesis and processing

## Abstract

Boron nitride nanotubes (BNNTs) have attracted attention for their predicted extraordinary properties; yet, challenges in synthesis and processing have stifled progress on macroscopic materials. Recent advances have led to the production of highly pure BNNTs. Here we report that neat BNNTs dissolve in chlorosulfonic acid (CSA) and form birefringent liquid crystal domains at concentrations above 170 ppmw. These tactoidal domains merge into millimeter-sized regions upon light sonication in capillaries. Cryogenic electron microscopy directly shows nematic alignment of BNNTs in solution. BNNT liquid crystals can be processed into aligned films and extruded into neat BNNT fibers. This study of nematic liquid crystals of BNNTs demonstrates their ability to form macroscopic materials to be used in high-performance applications.

## Abstract (web summary)

Boron nitride nanotubes (BNNTS) have only been shown to dissolve in chlorosulfonic acid (CSA) at low concentrations. Here the authors successfully demonstrate the formation of liquid crystals of BNNTs in CSA that can be used to produce macroscopically aligned neat fibers of BNNTs.

## Introduction

Boron nitride nanotubes (BNNTs) are high aspect ratio rod-like nanostructures a few nanometers in diameter and microns long^1^. Chemically, BNNTs are composed of alternating boron and nitrogen atoms in a hexagonally-bonded sheet, scrolled to form a seamless cylindrical structure that gives rise to several unique properties^2^. In addition to being mechanically strong^3,4^, BNNTs are thermally conductive^5^, electrically insulating^2^, neutron-shielding^6^, piezoelectric^7^, and thermally stable up to 900 °C in air^8,9^. These properties are desirable for many applications, including aerospace, electronics, and energy-efficient materials. However, the utility of BNNTs is not yet fully realized, because their remarkable properties have only been observed at the microscopic level^10^. Future improvements in material quality and processing techniques will enable high-performance neat BNNT articles with extraordinary properties for use in extreme environments.

The inherent properties of nanoscale building blocks can be translated to the macroscopic scale by controlling long-range ordering, as has been achieved with carbon nanotubes (CNTs)^11^, structural analogs of BNNTs that share many of their desirable properties, apart from superior thermal stability^12^. For example, closely packed and highly aligned CNT fibers yield high performance (e.g., tensile strength^13^ above 4 GPa and electrical conductivity^13^ above 10 MS/m) via multiple routes, including direct spinning^14,15^, wet spinning^16–18^, and carpet spinning^19,20^. Thus far, none of these methods has been effectively employed to produce ordered BNNT materials. Of these routes, wet spinning appears most easily adapted to processing BNNTs because it is independent of synthesis method. Yet, wet spinning requires a solvent for the nanotubes and preferably the formation of a nanorod liquid crystal. Dispersions of individualized BNNTs can be achieved using chlorosulfonic acid (CSA)^21^. Kleinerman et al. hypothesized that protonation of nitrogen atoms of the outer BNNT wall confers a net positive charge to their surface; positively charged BNNTs repel each other leading to their individualization, as is the case for CNTs^21^. However, the bond structure of BNNTs would localize protons on the nitrogen atoms—unlike in CNTs, where shared π electrons favor delocalization and hence further stabilization of positive charges when CNTs are dissolved in acids^22^. This less effective charge stabilization (and charge localization on the nitrogen atoms) is likely to lead to some residual long-range attraction of BNNTs in CSA (which is absent for CNTs in CSA^23^), consistent with the higher-than-predicted isotropic-nematic phase transitions observed in this work. Thus far, the formation of BNNT liquid crystals has not been attained, possibly because of sample impurities (e.g., hexagonal boron nitride (h-BN) and elemental boron^24^), which hinder the individualization and alignment of BNNTs. Previous work has suggested spontaneous ordering of DNA-BNNT hybrid systems, but aligned structures were only observed in dried films of DNA-wrapped BNNTs after solvent removal by filtration^25^ or evaporation^26^.

Here, we show that high-quality, purified BNNTs dissolve in CSA to form liquid crystals, including bipolar nematic tactoids, at concentrations as low as 170 ppm; these domains can be merged into bulk nematic domains using mild sonication. We image the BNNT liquid crystalline structure via cryogenic electron microscopy. We show that BNNT liquid crystals can be processed into aligned films and fibers using existing scalable techniques.

## Results and discussion

**Fig. 1:**
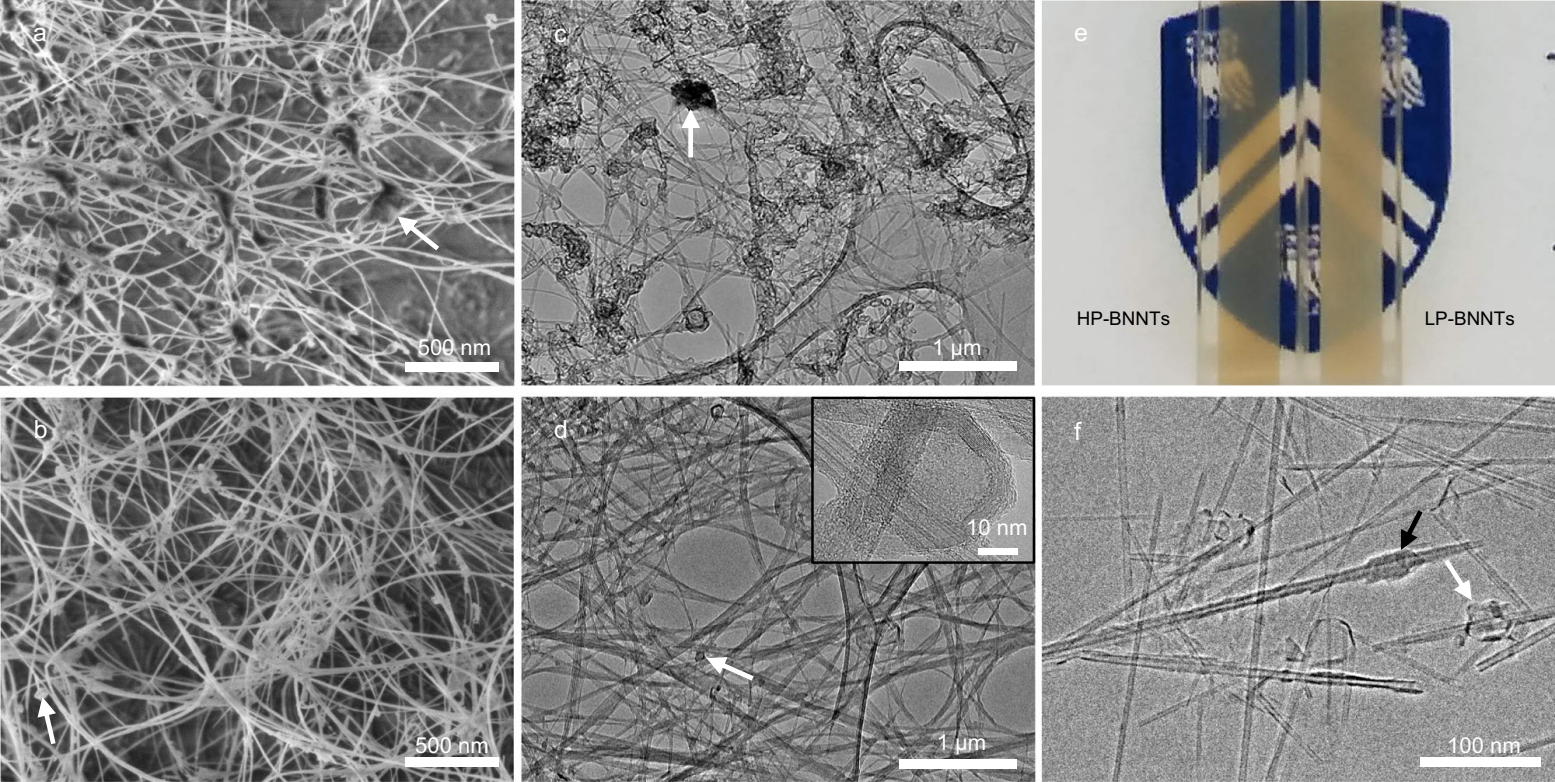
Purity assessment of lightly purified BNNTs (LP-BNNTs) and highly purified BNNTs (HP-BNNTs). **a** Scanning electron microscopy (SEM) of LP-BNNTs. **b** SEM of HP-BNNTs. **c** Transmission electron microscopy (TEM) of LP-BNNTs on perforated carbon grid. **d** TEM of HP-BNNTs on perforated carbon grid with inset TEM image of residual contaminants in HP-BNNTs. A selected area fast Fourier transform of the inset image indicates an interlayer spacing of 0.337 nm, the same as for h-BN (Supplementary Fig. 4). Electron microscopy shows a reduction in non-nanotube structures upon more purification. Arrows indicate hexagonal boron nitride (h-BN) contaminants in both LP-BNNTs and HP-BNNTs. **e** Photograph of solutions of (left) HP-BNNTs and (right) LP-BNNTs in chlorosulfonic acid (CSA) at 1700 ppmw in 4 mm path length quartz cuvettes placed in front of the Rice University shield. Scattering of the LP-BNNT solution occludes details in the design. **f** Cryogenic-TEM of 4000 ppmw HP-BNNTs in CSA showing amorphous material covering BNNTs (black arrow), and faceted structures (white arrow).

We synthesize the BNNTs via the high-temperature-pressure method (HTP)^24^ and purify them to remove residual elemental boron, producing “lightly processed” BNNTs (LP-BNNTs). LP-BNNTs are further purified to remove non-nanotube BN, producing “highly processed” BNNTs (HP-BNNTs)—see “Methods”. Thermogravimetric analysis (TGA) of LP-BNNTs and HP-BNNTs in air (Supplementary Fig. 1) shows no mass gain from oxidation below 900 °C, indicating the absence of elemental boron impurities. The 35% mass increase at 1100 °C due to the oxidation of boron nitride is consistent with TGA from previous reports of pristine BNNTs^27^. Because TGA does not distinguish between the oxidation of BNNTs and other boron nitride allotropes, scanning electron microscopy (SEM) and transmission electron microscopy (TEM) are used to assess relative BNNT purities. Figure 1a, c shows that LP-BNNTs samples contain agglomerates of h-BN (white arrows) that envelop overlapping BNNT bundles to create an apparent crosslinked BNNT network. The HP-BNNT samples (Fig. 1b, d) have a lower concentration of smaller h-BN aggregates (white arrows). TEM shown in Fig. 1d reveals that the few remaining non-nanotube structures in HP-BNNTs are few-layer stacks of h-BN. Consistent with literature values^28^, the h-BN stack exhibits an interlayer spacing of 0.337 nm (Supplementary Fig. 2), determined by selected area fast Fourier transform.

Purified BNNTs spontaneously dissolve in CSA upon high-shear mixing to form homogenous liquids. LP-BNNTs in CSA (Fig. 1e, right) appear white and opaque, likely from scattering of micron sized aggregates of h-BN and BNNTs (visible in Fig. 1a, c) that do not dissolve well. Conversely, HP-BNNT solutions (Fig. 1e, left) typically have a peach color and are more transparent to the eye at the same concentration due to reduced optical scattering from individualization, owing to a superior purity. UV–visible and Fourier transform infrared (FTIR) spectroscopy indicate that dissolution in CSA does not chemically alter BNNTs (Supplementary Figs. 3 and 4). Cryogenic-TEM (Cryo-TEM) of low-concentration HP-BNNT solutions (Fig. 1f) shows individualized BNNTs in CSA. Cryo-TEM also reveals that trace contaminants in HP-BNNTs (black and white arrows) envelop short segments of individual BNNTs. HP-BNNT diameter and length are characterized by AFM (Supplementary Fig. 5a–d), diameter and number of walls by TEM (Supplementary Fig. 5e–g), and aspect ratio via capillary thinning extensional rheometry^29^ (Supplementary Fig. 6). The results summarized in Table 1 show that all the techniques yield consistent results (within measurement error), with a typical aspect ratio of ~300 for HP-BNNT.

**Table 1:** Summary of bootstrapped HP-BNNT statistics.

	Rheometry/TEM	AFM
Diameter (nm)	5.8 ± 0.3	5.6 ± 0.2
Length (μm)	1.65 ± 0.29	1.54 ± 0.06
Aspect ratio (L/D)	257 ± 46	294 ± 14

**Fig. 2:**
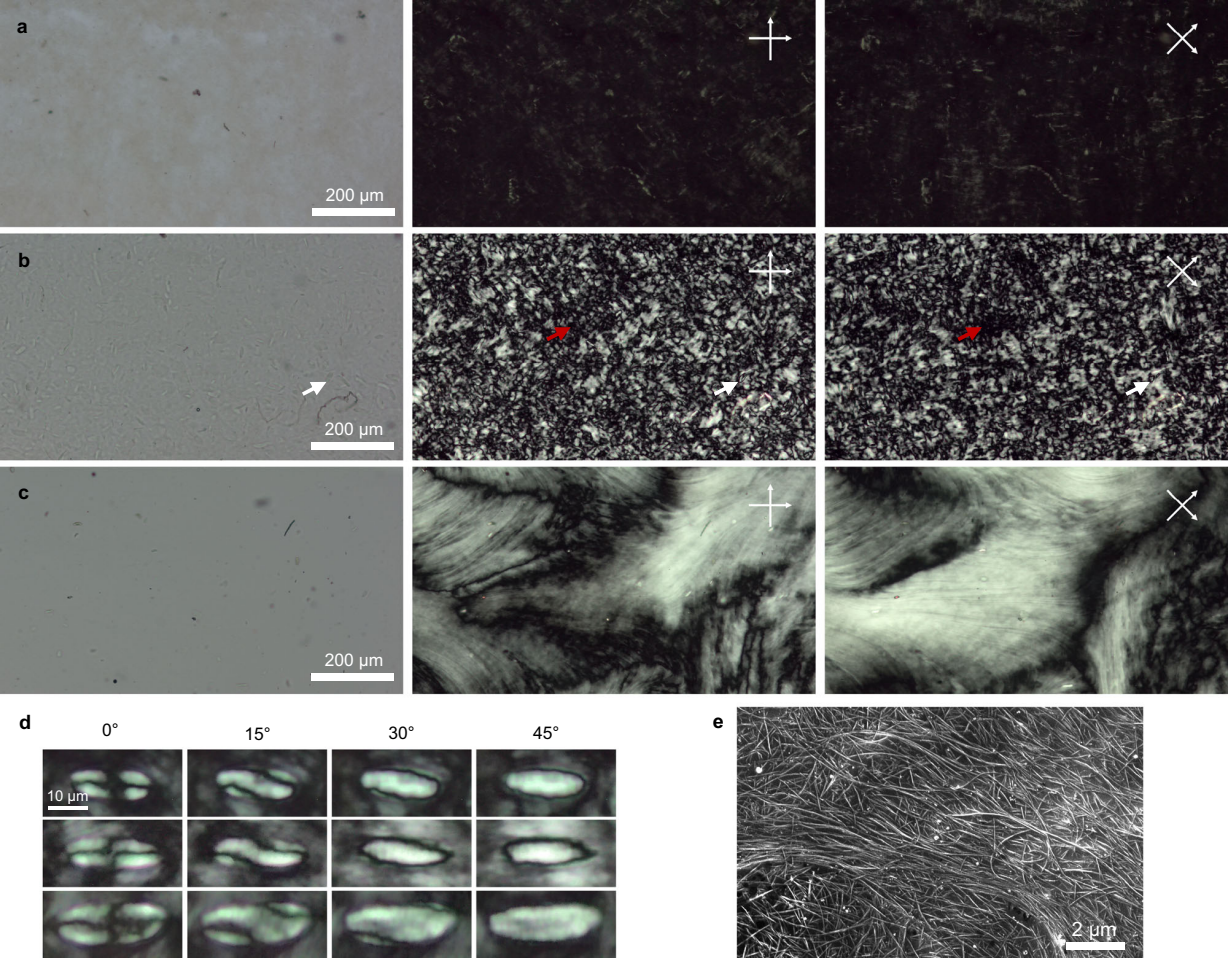
Liquid crystals of BNNTs. Polarized light microscopy (PLM) of BNNTs in chlorosulfonic acid (CSA) in flame-sealed 1 mm × 0.1 mm rectangular capillaries (**a**–**c**): unpolarized transmitted light and polarized light images with polarizer/analyzer at 0°/90° and 45°/135°, as depicted by the white crossed arrows. **a** Solution of lightly purified BNNTs (LP-BNNTs) at 7000 ppmw showing an isotropic solution with few birefringent domains. **b** Solution of highly purified BNNTs (HP-BNNTs) at 7260 ppmw showing 20 μm regions of strong birefringence. White arrows in **b** highlight the same elliptical structure in all three images. Red arrows highlight the same isotropic region in both polarized images. **c** Solution of HP-BNNTs at 7260 ppmw (the same solution as **b**) after ~5 s of bath sonication of the filled capillary. **d** High magnification PLM images after dilution of the solution in **b** to 3000 ppmw showing three different tactoids between crossed-polarizer and analyzer at various angles. The scale bar is 10 μm for all tactoid images. **e** Cryogenic scanning electron micrograph of BNNTs in CSA at 8000 ppmw showing aligned and isotropic regions in a biphasic BNNT solution.

Polarized light microscopy (PLM) images of BNNT solutions in sealed capillaries reveal important information about the solution quality such as degree of dissolution and anisotropy. LP-BNNTs at 7000 ppm by weight (ppmw) (Fig. 2a) have a gel-like behavior and show little birefringence, indicating no molecular orientational ordering, likely because BNNTs are trapped in the h-BN-induced network visible in Fig. 1a, c. Conversely, solutions of HP-BNNTs (Fig. 2b) display a brightly birefringent Schlieren texture, a hallmark of nematic liquid crystals^30^. The presence of areas that remain dark for all crossed-polarizer angles (red arrows) suggests that the solution is biphasic (isotropic-nematic). Brief bath sonication of these filled capillaries promotes the merging of the nematic regions into larger domains that span the 1 mm width of the capillary (Fig. 2c). The unpolarized light image of HP-BNNTs (Fig. 2b, left panel) shows 20 μm long ellipsoidal regions dispersed throughout the sample. The white arrows in Fig. 2b point to the same ellipsoidal structure. At high magnifications (Fig. 2d), the ellipsoids display the birefringence pattern of bipolar tactoids, small spindle-shaped nonequilibrium nematic droplets surrounded by isotropic media that form in concentrated solutions of elongated particles^31,32^. While the tactoids in unsonicated capillaries are stable for over 1 year, the sonicated liquid crystalline domains are stable in sealed capillaries for at least 6 months, limited by CSA degradation due to the slow ingress of moisture from sealed capillary ends that can be damaged by sonication. Likely, sonication provides enough energy to overcome the kinetic barriers that prevent the merging of tactoids. Tactoids are observed in solutions of HP-BNNTs at all studied concentrations, from 50 ppmw to 7260 ppmw (Supplementary Fig. 7a, b). The isotropic cloud point for these BNNTs was determined to be 170 ppmw (220 ppm by volume). This determination was made using images of capillary-sonicated solutions in Supplementary Fig. 7c, d so that BNNTs in tactoids persisting after dilution would fully disperse. Interestingly, the isotropic cloud point for these BNNTs is an order of magnitude lower than what would be expected for CNTs of similar aspect ratio^29^. This is consistent with less effective charge stabilization relative to CNTs, resulting in residual attraction and lowered isotropic cloud points, as observed in CNT solutions with decreasing acid strength^23,33^. Nematic domains of HP-BNNTs (8000 ppmw = 0.8 wt%) are directly visualized in Fig. 2e by cryogenic scanning electron microscopy (cryo-SEM), which shows an aligned domain surrounded by isotropic BNNTs, consistent with the non-birefringent areas present in Fig. 2b. No tactoids were observed in cryo-SEM, but this is likely because such structures would have to lie along the fracture plane of the vitrified solution with the proper orientation to be visualized. Sonication of HP-BNNT solutions prior to loading into capillaries can in some cases break up tactoid domains while retaining the Schlieren texture observed in Fig. 2b. Although the impurities in LP-BNNTs solutions inhibit BNNT dissolution and the formation of tactoids, capillary sonication results in the same long-range alignment as in HP-BNNT solutions, but with a significant quantity of undispersed particles present (Supplementary Fig. 8).

**Fig. 3:**
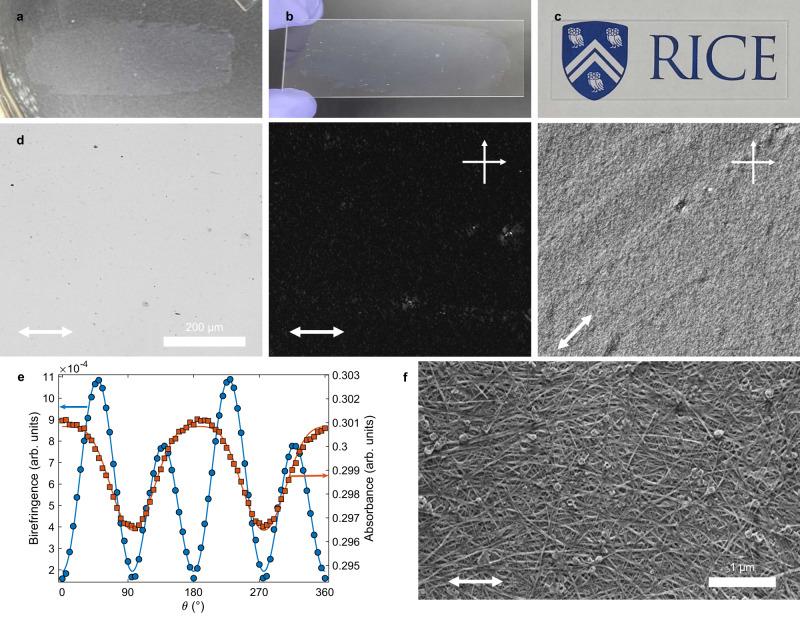
Aligned films of BNNTs. **a** Photograph of a BNNT film made from a ~1 wt% solution of highly purified BNNTs (HP-BNNTs) floating on water with its original structure intact. **b** Photograph of the same film as in **a** redeposited onto a new glass slide and held at an angle to observe the film more easily. **c** Photograph of the BNNT film from **b** on the glass slide laid over the Rice shield, demonstrating high transparency. **d** Polarized light microscopy (PLM) images of the BNNT film on a glass slide: unpolarized transmitted light image and polarized light images with polarizer/analyzer at fixed at 0°/90°. The film was rotated to 45° for the last polarized light image and the double-sided white arrows in the lower left corners of the PLM images indicate the orientation of the shear axis for the film. **e** Plot of HP-BNNT film birefringence intensity normalized by incident light intensity (circles) and film absorbance (squares) against stage rotation angle. Birefringence intensity and absorbance are both plotted with model fits with coefficients of determination (*r*^2^) of 0.996 and 0.991, respectively. **f** Scanning electron micrograph of the BNNT film on glass. The double-sided white arrow indicates the shear axis for the film on SEM micrograph.

The spontaneous alignment of BNNTs in liquid crystalline solutions is advantageous for the assembly of these building blocks into ordered macroscopic materials. Here, we are using lower concentration biphasic solutions in order to obtain processable volumes with the amount of HP-BNNT material available at this time. The lack of a fully liquid crystalline solution does not preclude the possibility of producing solid BNNT assemblies, because controlled flow induces alignment, even in an isotropic solution of rigid rods^34^—in fact, extensional flow was recently used to form continuous macroscopic fibers of BNNT/polyvinyl alcohol^35^ by adapting the spinning technique originally developed for making CNT/PVA fibers^16^. We produce aligned BNNT films of HP-BNNTs by manually shearing a small aliquot of their solution in CSA between two glass slides. The shear forces align nematic domains along the shear direction. Coagulation in acetone immediately after shearing preserves the ordered structure by quickly removing CSA from the BNNTs, which adhere to the glass slide. Fully coagulated films can be lifted from the glass slide and floated on the surface of a bath of water (Fig. 3a). The films can then be redeposited onto other structures, such as another glass slide (Fig. 3b). These thin BNNT films are highly transparent to visible light, uniform, and free of haze, as displayed by the sharp image of the Rice University shield below the film (Fig. 3c). PLM of these films (Fig. 3d) shows a dark field of view for crossed-polarizer orientations of 0°/90° and bright field of view for a 45°/135°, indicating BNNT alignment along the direction of shear (0°). The periodic birefringence intensity and absorbance can be fit to classical equation forms for anisotropic films, sin^2^(*θ*)cos^2^(*θ*) and sin^2^(*θ*), respectively^36^. Good fits (Fig. 3e) to the expected birefringence and absorbance indicate that the HP-BNNT film is aligned along the shear direction—although absorbance differences are minimal, because the visible light absorbance of BNNTs is low both for parallel and perpendicular orientations between the BNNT and the light polarization. The reduced birefringence intensities at 135° and 315° are likely due to a twist in the nematic director of the film^36^. The dichroic ratio, determined from the birefringence intensity in Fig. 3e, can be used^37^ to estimate the film order parameter *S*_bir_ = 0.56. Alignment of BNNT bundles can be observed by SEM, which shows mild alignment along the shear direction on the top layer of BNNTs (Fig. 3f).

**Fig. 4:**
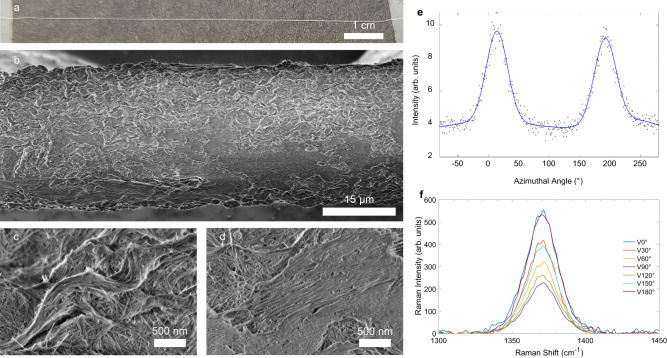
Solution-spun fibers of BNNTs. **a** Photograph of typical segment of fiber made from highly purified BNNTs taped to a benchtop under tension. **b** Scanning electron microscopy (SEM) showing a 27 μm diameter fiber with wrinkled and smooth areas of the fiber skin. **c** SEM showing mild alignment of BNNTs along the wrinkle contours. **d** SEM of a smooth area of the fiber surface showing strong BNNT alignment along the fiber axis. **e** Wide-angle X-ray scattering of BNNT fiber (black dots) plotted against azimuthal angle shows two peaks from the fiber fit with a Gaussian curve (blue line) with full-width at half maximum (FWHM) of 42°. **f** Raman spectra of BNNT fiber with V0°, V30°, V60°, V90°, V120°, V150°, and V180° polarizer/analyzer modes. Laser excitation was given by a 785 nm laser. A sharp peak at ∼1370 cm^−1^ is characteristic of BNNTs.

Liquid crystals are ideal precursors for aligned BNNT fibers. We produce fibers of neat HP-BNNTs via wet solution spinning, similar to other acid solution spinning processes^38,39^. The ~1 wt% HP-BNNT solution, or dope, is extruded vertically from a syringe through a 150 μm spinneret into acetone. Acetone coagulates the extruding filament into a solid fiber by extracting and reacting with CSA (Fig. 4a). Figure 4b shows SEM of typical fibers, with an average diameter of 27 μm ± 1 μm variation along the fiber length. Higher magnifications in SEM reveal a wrinkled skin with mild axial alignment of BNNTs along most of the fiber. The uneven surface morphology is likely a result of collapse induced by rapid evaporation of the coagulant from the BNNT filament after removal from the coagulant bath with no applied draw during extrusion. Closer inspection of the surface wrinkles shows BNNT alignment along their contours (Fig. 4c). Elsewhere are isolated domains of highly aligned BNNTs oriented along the fiber axis (Fig. 4d). Wide-angle X-ray scattering (Fig. 4e) of typical BNNT fibers shows axial alignment. The full-width-half-max (FWHM) of 42° indicates that the BNNT fiber has somewhat higher alignment than early CNT fibers^40^, which had a FWHM of ~60°. Polarized Raman spectra of that same BNNT fiber show a strong peak of BNNT at around 1370 cm^−1^ (Fig. 4f), which corresponds to the transverse optical A1 vibrational mode^41,42^. The intensity of the 1370 cm^−1^ band is shown to be polarization-dependent, which is consistent with BNNT alignment. Figure 4f shows that the maximum intensity of the characteristic peak at 1370 cm^−1^ is under the parallel polarization modes (V0° and V180°) and the minimum is under the perpendicular polarization (V90°) mode, suggesting a two-fold symmetry. The peak intensity ratio of V0°:V90° is estimated as 2.4:1, consistent with mild BNNT alignment along the fiber axis. This ratio is somewhat lower than the Raman peak ratio of 5.1:1 observed in early CNT fiber^40^. The strongest BNNT fibers show an average (over twelve measurements) tensile strength of 15.7 MPa ± 1.6 MPa and Young’s modulus of 1.46 GPa ± 0.18 GPa, based on a diameter of 27 μm and circular cross-section. The BNNT fiber properties reported here are encouraging, given the similarities between BNNTs^4^ and CNTs^43^. In fact, these initial results are comparable to those of early solution-spun HiPco CNT fiber, which had a strength of ~1 to 5 MPa and modulus of ~0.1 to 1 GPa (see Chapter 3 of ref. ^44^). The performance of early HiPco CNT fiber was limited by material impurity, but was later improved by two orders of magnitude by optimizing HiPco material processing^38^. Later, CNT fiber performance was further improved by a factor of ~50 by combined advances in CNT quality and spinning^18^.

In conclusion, we have shown that purified few-walled BNNTs are soluble in CSA and can be processed into macroscopic materials. BNNTs form nematic liquid crystals in CSA, including tactoids in biphasic solutions at the concentrations we tested (~1 wt%). We measured the aspect ratio of BNNTs in bulk using capillary thinning rheometry, which provides an important structural parameter (similar to intrinsic viscosity in polymers), because the mechanical and thermal properties of macroscopic BNNT materials should scale with aspect ratio, as shown for CNTs^18^. We expect BNNTs to form fully liquid crystalline solutions, and the concentration of this transition is expected to decrease with higher aspect ratio^45^ and higher purity. Thus, the challenge of forming fully liquid crystalline BNNT solutions at processable concentrations lies in increasing both of these material parameters. We process BNNT liquid crystals into aligned films and neat BNNT fibers with encouraging structural order. Currently, the properties of these BNNT films and fibers are likely limited by a combination of aspect ratio, solution quality, and processing parameters. Further improvements in material synthesis, purification, and mapping of the liquid crystalline phase diagram will yield optimal processing dopes. The nematic transition for solutions of CNTs has been observed through rheological characterization of CNT-superacid solutions^46^, and such experiments could help identify this transition for BNNT solutions. Fully liquid crystalline, higher concentration dopes will be explored in future experiments as HP-BNNTs become more broadly available. Such improved dopes will allow spinning in a broader parameter space with stable filament draw, uniform diameter, higher BNNT alignment, and higher packing density, all of which are directly correlated with macroscopic fiber properties^18^. This work demonstrates a scalable methodology for producing ordered BNNT materials that retain their extraordinary properties in extreme environments.

## Methods

### Synthesis and purification of BNNTs

BNNTs for this research were synthesized by BNNT LLC (Newport News, VA), using the HTP method^24^. The as-synthesized samples (product code SP10) were then purified to remove elemental boron^47^, yielding LP-BNNTs (product code SP10R). LP-BNNTs include non-nanotube boron nitride species, which can be removed by high-temperature steam purification^27^. LP-BNNTs were further purified by a high-temperature steam purification process to remove non-nanotube BN species, yielding HP-BNNTs (product code SP10RX). TGA was performed using a Mettler Toledo TGA/DSC 3+ in air with a 10-min hold at 110 °C and a temperature ramp of 10 °C/min to 1100 °C for all samples.

### Solution preparation and spectroscopy

The BNNTs were dissolved in CSA at a concentration of ~1% by weight. Pure (99.9%) CSA was used as received from Sigma-Aldrich. The vial containing BNNTs and CSA was first vigorously shaken by hand to break apart the BNNT “puff ball,” prior to mixing for 40 min with a FlackTek DAC 600 SpeedMixer forming a viscous solution. UV–vis spectra of the BNNTs dissolved in CSA and dispersed in 1% by weight aqueous sodium dodecyl sulfate (SDS) were collected using a Shimadzu UV-1800 spectrophotometer. Quartz cuvettes with PTFE caps and path lengths of 1 mm and 1 cm were used for CSA and SDS samples, respectively. BNNT solutions in CSA were quenched and rinsed with deionized water until a neutral pH was reached. The BNNTs were then dried to collect FTIR spectra. A Nicolet iS50 instrument was used with a diamond ATR accessory, averaging 32 scans at 4 cm^−1^ resolution.

### Rheology and aspect ratio determination of BNNTs

Viscosity-averaged aspect ratios were estimated using a custom-built capillary thinning rheometer, described by ref. ^29^. The extensional viscosity is determined by the rate-of-change of the filament diameter. Supplementary Fig. 6 shows the final frames of filament thinning that were recorded during the experiment. An extensional viscosity of 0.4 Pa s was determined for the 7260 ppmw solution. A density of 1.50 g/cm^3^ was used for the HP-BNNTs to calculate the viscosity-averaged aspect ratio. This density was calculated assuming that a single-walled BNNT has the same areal density as a single sheet of h-BN (1318 m^2^/g), following along with similar calculations for graphene^48^. The density of HP-BNNTs was calculated using the average number of walls (3.5) and outer diameter (5.8 nm ± 0.3 nm), while also assuming with a BN bond length of 1.446 Å, and an interlayer spacing of 3.331 Å (refs. ^28,49^).

### BNNT films and fibers

Shear-aligned films were produced according to the method described by ref. ^50^, using acetone as the coagulant. Just prior to shearing the solution, a powered 20 kHz Qsonica Q55 tip sonicator was lightly brushed along the outer surface of the top glass, scratching the glass and merging tactoid domains. The top slide was then rapidly sheared across the bottom slide and immediately plunged into an acetone coagulant bath. Shear-aligned films were annealed in air at 400 °C for 20 min to improve electron imaging quality by removing residual acid and organic molecules from coagulation. BNNT fibers were produced by extruding BNNT solution through a 150 μm spinneret into a coagulation bath of acetone, based on methods described by ref. ^18^. Fiber was extracted from the bath and allowed to dry under ambient conditions overnight, before tensile testing on an ARES-G2 rheometer, using 4 mm fiber segments.

### Light microscopy

Portions of HP-BNNT and LP-BNNT CSA solutions were loaded and flame-sealed into glass capillaries with an inner width of 100 μm, which were then flame-sealed. Some sealed capillaries were sonicated by submerging them into a Branson 1800 bath sonicator at 40 kHz for ~5 s. Light microscopy was performed with a Zeiss Axioplan 2 microscope equipped with a Zeiss Axiocam 208 camera. The contrast for images of sealed capillaries was adjusted in MatLab and ImageJ. Each set of PLM images received the same contrast adjustments. Images of the BNNT film that were used to study birefringence and absorption were taken with the camera operating in black-and-white mode without contrast adjustments. Intensities for each rotation angle were determined by averaging the pixel values across the entire field of view (~1 mm × 0.65 mm). The incident light intensity was determined by capturing an image with no sample and only the polarizer inserted. All images for this analysis were taken using the same microscope illumination settings and intensity values were normalized by camera exposure time.

### Atomic force microscopy

Samples for atomic force microscopy (AFM) were prepared from solutions diluted to ~1 ppmw and drop-casted onto a freshly cleaved mica surface, preheated to 400 °C using a hot plate. Diethyl ether was used to remove residual acid from the mica before drying the sample with room temperature air flow, followed by oven treatment at 100 °C for an hour. The AFM measurements were performed with a Nanoscope IIIa scanning probe microscope controller from Digital Instruments operated in the tapping mode. Micrographs were processed in the Gwyddion software package, and the height profiles and length of 100 randomly selected individual nanotubes were collected.

### Electron microscopy

SEM of dry material was performed using an FEI Helios NanoLab 660 SEM/FIB. Shear-aligned films were imaged directly on glass, and the as-produced material was imaged on carbon tape. SEM samples received no conductive layer prior to imaging. An accelerating voltage of ~1 kV and working distance of ~3.5 mm was used for SEM characterization.

TEM and Cryo-TEM of BNNT-CSA solutions were performed by a Thermo Fisher (formerly FEI) Talos 200C high-resolution TEM at an accelerating voltage of 200 kV. Cryo-specimens were maintained below −175 °C in the microscope using a Gatan 626 cryo-holder, and imaged in the low-dose-imaging mode to reduce electron-beam radiation damage. Images were recorded digitally by an FEI Falcon III direct-imaging camera and the TIA software, with the help of the “Volta phase-plate” (FEI) to enhance image contrast.

Cryo-SEM of BNNT-CSA solutions was performed by a Zeiss Ultra Plus HR-SEM equipped with a Leica VT100 cold-stage system. Specimens were maintained at −145 °C, and imaged without coating at a low acceleration voltage of 0.6 kV to achieve a state of charge balance, avoiding specimen charging and imaging artifacts^51^. Micrographs were taken at short working distance (3.3–3.8 mm) with an in-the-column secondary electron detector. Cryo-TEM and cryo-SEM specimens were prepared in a controlled environment vitrification system^52^, continuously purged with dry N_2_ to prevent superacid reaction with moisture. For cryo-TEM specimens, a drop was applied onto a perforated carbon film supported on a 3 mm copper TEM grid. The drop was blotted with a fiberglass filter-paper to form a thin film (<300 nm) and vitrified by plunging into liquid nitrogen. Cryo-SEM specimens were prepared by dipping a 3 mm grid in the solution and placing it between two gold planchettes. The specimen was plunged into liquid nitrogen with dedicated tweezers^53^, and freeze-fractured in a BAF060 unit (Leica AG, Liechtenstein) at −167 °C.

### Raman spectroscopy

Raman spectra were obtained with a DXR2 Raman microscope system (Thermo Fisher Scientific) equipped with a laser excitation of 785 nm under ×50 objective. The BNNT fiber samples were placed onto a glass slide, and analyzed within the Raman shift range of 200–3400 cm^−1^. The Raman microscope was operated with the following experimental parameters: excitation laser wavelength (785 nm), laser intensity (30 mW), and aperture type (50 µm pinhole aperture). The spectral background was corrected using smart subtraction offered by the DXR2 Raman microscope to remove interference caused by fluorescence. The alignment of the BNNT fiber was determined by recording a Raman spectrum every 30° from 0° to 180° between the incident polarization and the fiber axis. While the polarizer was set vertically, Raman spectra were collected from BNNT fiber in the order of V0° (parallel polarization), V30°, V60°, V90° (perpendicular polarization), V120°, V150°, and V180° polarizer/analyzer modes.

### Wide-angle X-ray scattering

Wide-angle X-ray diffraction was performed using a small/wide-angle diffractometer (Molecular Metrology SAXS system), equipped with a sealed microfocus tube (MicroMax-002 + S) emitting Cu Kα radiation (*λ* = 1.542 Å), two Göbel mirrors, and three pinhole slits. The generator is powered at 45 kV and 0.8 mA. The scattering patterns were recorded by a 15 × 15 cm two-dimensional imaging plate (BAS-IP-MS, FUJIFILM), positioned about 6 cm behind the sample. Exposition time was about 18 h. The single fiber sample was fixed on a two-dimensional holder perpendicular to the beam, and measured under vacuum at ambient temperature. The imaging plate was scanned by a Fluorescent Image Analyzing System (FLA-7000), and analyzed by FLA-7000 ImageReader software with 100 μm resolution.

## Supplementary information

Supplementary Information

## Data Availability

The main data that support the findings of this study are available in the article and Supplementary Information. Raw data are available from the corresponding author upon reasonable request.
