# Supplementary material for: Liquid crystals of neat boron nitride nanotubes and their assembly into ordered macroscopic materials
Source: Nat Commun. 2022 Jun 7;13:3136. doi: 10.1038/s41467-022-30378-5 (PMC9174261; doi:10.1038/s41467-022-30378-5)
Supplement: Supplementary file 1 — Supplementary Information [file 41467_2022_30378_MOESM1_ESM.pdf]

Supplementary Information for

# Liquid Crystals of Neat Boron Nitride Nanotubes and their Assembly into Ordered Macroscopic Materials

Cedric J. S. Ginestra[1], Cecilia Martínez-Jiménez[2], Asia Matatyaho Ya'akobi[3], Oliver S. Dewey[1], Ashleigh D. Smith McWilliams[2], Robert J. Headrick[1,2], Jesus A. Acapulco[1], Lyndsey R. Scammell[4], Michael W. Smith[4], Dmitry V. Kosynkin[1], Daniel M. Marincel[1,5], Cheol Park[6], Sang-Hyon Chu[7], Yeshayahu Talmon[3], Angel A. Martí[2,8,9,10]*, and Matteo Pasquali[1,2,8,10]*

[1]Department of Chemical and Biomolecular Engineering, Rice University, 6100 Main Street, MS 369, Houston, Texas 77005, United States

[2]Department of Chemistry, Rice University, 6100 Main Street, MS 369, Houston, Texas 77005, United States

[3]Department of Chemical Engineering and the Russell Berrie Nanotechnology Institute (RBNI), Technion-Israel Institute of Technology, Haifa 3200003, Israel

[4]BNNT Materials, LLC, 300 Ed Wright Lane Suite A, Newport News, Virginia 23606, United States

[5] Department of Physics and Optical Engineering, Rose-Hulman Institute of Technology, 5500 Wabash Ave, CM 169, Terre Haute, Indiana 47803, United States

[6]Advanced Materials and Processing Branch, NASA Langley Research Center, Hampton, Virginia 23681, United States

[7]National Institute of Aerospace, 100 Exploration Way, Hampton, Virginia 23666, United States

[8]Department of Materials Science & NanoEngineering, 6100 Main Street, MS 369, Houston, Texas 77005, United States

[9]Department of BioEngineering, 6100 Main Street, MS 369, Houston, Texas 77005, United States

[10]The Smalley-Curl Institute, Rice University, 6100 Main Street, MS 369, Houston, Texas 77005, United States

*amarti@rice.edu; mp@rice.edu

Thermogravimetric analysis (TGA) of LP-BNNTs and HP-BNNTs in Supplementary Fig. 1 shows no residual boron after the two respective purifications. UV-vis spectroscopy of CSA solutions of HP-BNNTs and LP-BNNTs are shown in Supplementary Figs. 3a and 3b. Extinction coefficients at 350 nm (Supplementary Fig. 3c) are determined to be $9.87\times10^{-3}$ mL·μg$^{-1}$·cm$^{-1}$ for LP-BNNTs and $3.68\times10^{-3}$ mL·μg$^{-1}$·cm$^{-1}$ for HP-BNNTs. The larger extinction coefficient for LP-BNNTs quantifies the higher degree of scattering. These extinction coefficients can be used to determine solution concentrations. UV-vis spectra of recovered (previously dissolved in CSA) HP-BNNTs and LP-BNNTs in 1 wt% aqueous sodium dodecyl sulfate (SDS) solutions show maximum absorbances at 204 nm. The Tauc plot in Supplementary Fig. 3d is used to determine optical band gaps for HP-BNNTs and LP-BNNTs of 5.79 eV and 5.67 eV, respectively, which are consistent with the theoretical value of 5.5 eV (ref. [1]). The slightly higher band gap of HP-BNNTs relative to LP-BNNTs is consistent with band gap measurements of individual BNNTs and h-BN particles by electron energy loss spectroscopy[2], possibly indicating that the value reported here is sensitive to the degree of h-BN contamination. FTIR spectroscopy of recovered HP-BNNTs and LP-BNNTs (Supplementary Fig. 4) shows the B-N bending mode at 802 cm$^{-1}$ and B-N stretching at 1350 cm$^{-1}$ (ref. [3]), indicating that the chemical nature of BNNTs is maintained, even after purification, dispersion, quenching, and recovery from CSA.

TEM images of 106 BNNTs are analyzed for diameters and number of walls. The outer BNNT diameter by TEM is paired with aspect ratio measurements from extensional rheometry to determine length, and the number of walls is used to determine the mass density. Atomic force microscopy (AFM) images of 100 BNNTs are analyzed for BNNT diameters and lengths (Supplementary Fig. 5) as an independent measure of BNNT aspect ratio to validate the combined TEM and rheometry results. TEM and AFM data are resampled with replacement according to the

bootstrap method to determine more accurate estimates of variance for these data. The bootstrapped variable statistics approach the population statistic values in the limit of an infinite number of resampling, assuming that the sample is an unbiased representation of the population[4,5]. The size of each resampled group, $n$, was equal to the size of the parent data set, $N$, and each data set was sampled $10^6$ times.

HP-BNNT solutions are characterized via capillary thinning extensional rheometry (Supplementary Fig. 6), in which a liquid bridge of BNNT solution placed between two pistons is suddenly stretched in front of a high-speed camera. The rate of decrease of the liquid filament diameter is used to determine an extensional viscosity of the solution, which can be used to estimate the aspect ratio of rod-like nanoparticles[6]. HP-BNNT solutions behave as homogeneous liquids, similar to solutions of CNTs in CSA[7].

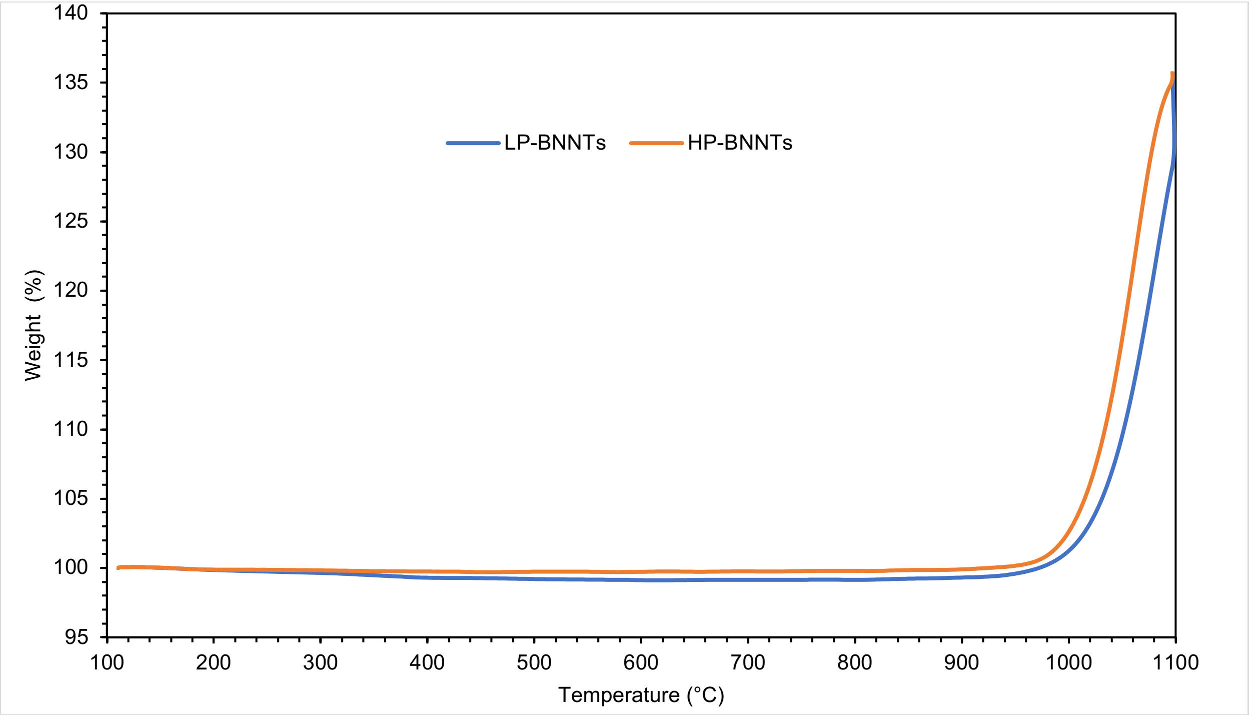


**Supplementary Fig. 1 Thermogravimetric analysis (TGA).** TGA of lightly purified BNNTs (LP-BNNTs) and highly purified (HP-BNNTs) was performed in dry air at 10°C/min to 1100°C with a 10-minute hold at 110°C to ensure samples were dry.

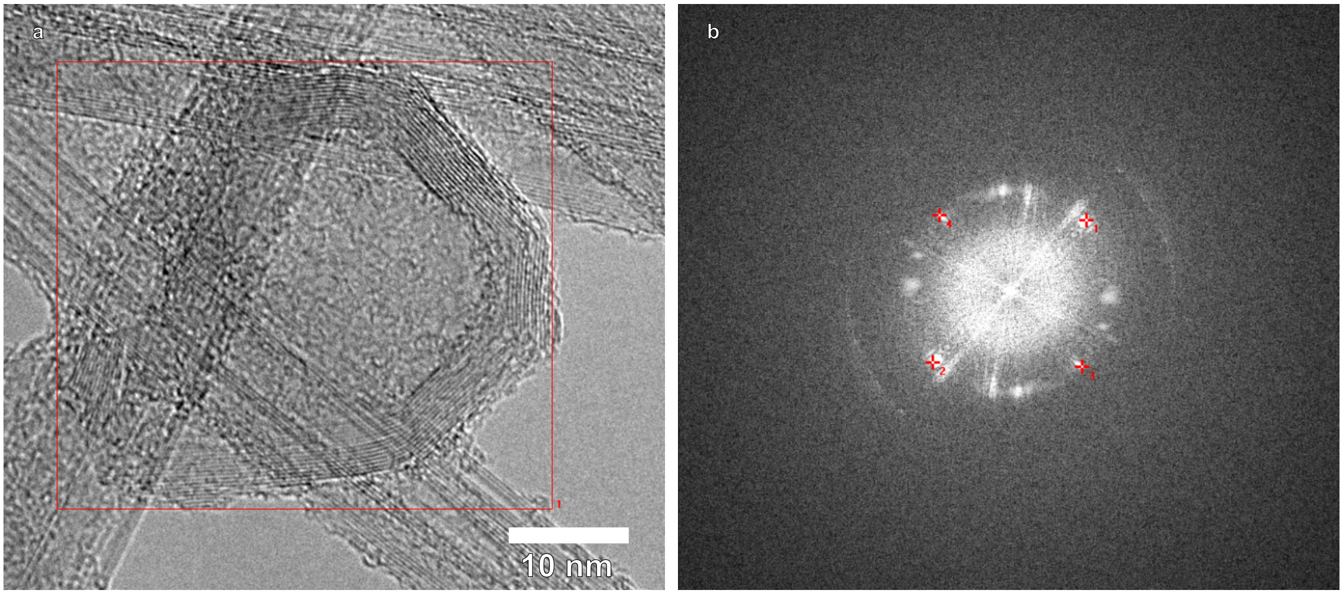


**Supplementary Fig. 2 HP-BNNT impurities. a** Transmission electron microscopy of a hexagonal boron nitride (h-BN) particle adhered to highly purified BNNTs with selected area outlined in red. **b** Selected area fast Fourier transform indicating an inter-layer spacing 0.337 nm. The few h-BN impurities that remain do not couple BNNTs together, but could negatively impact BNNT alignment and packing density.

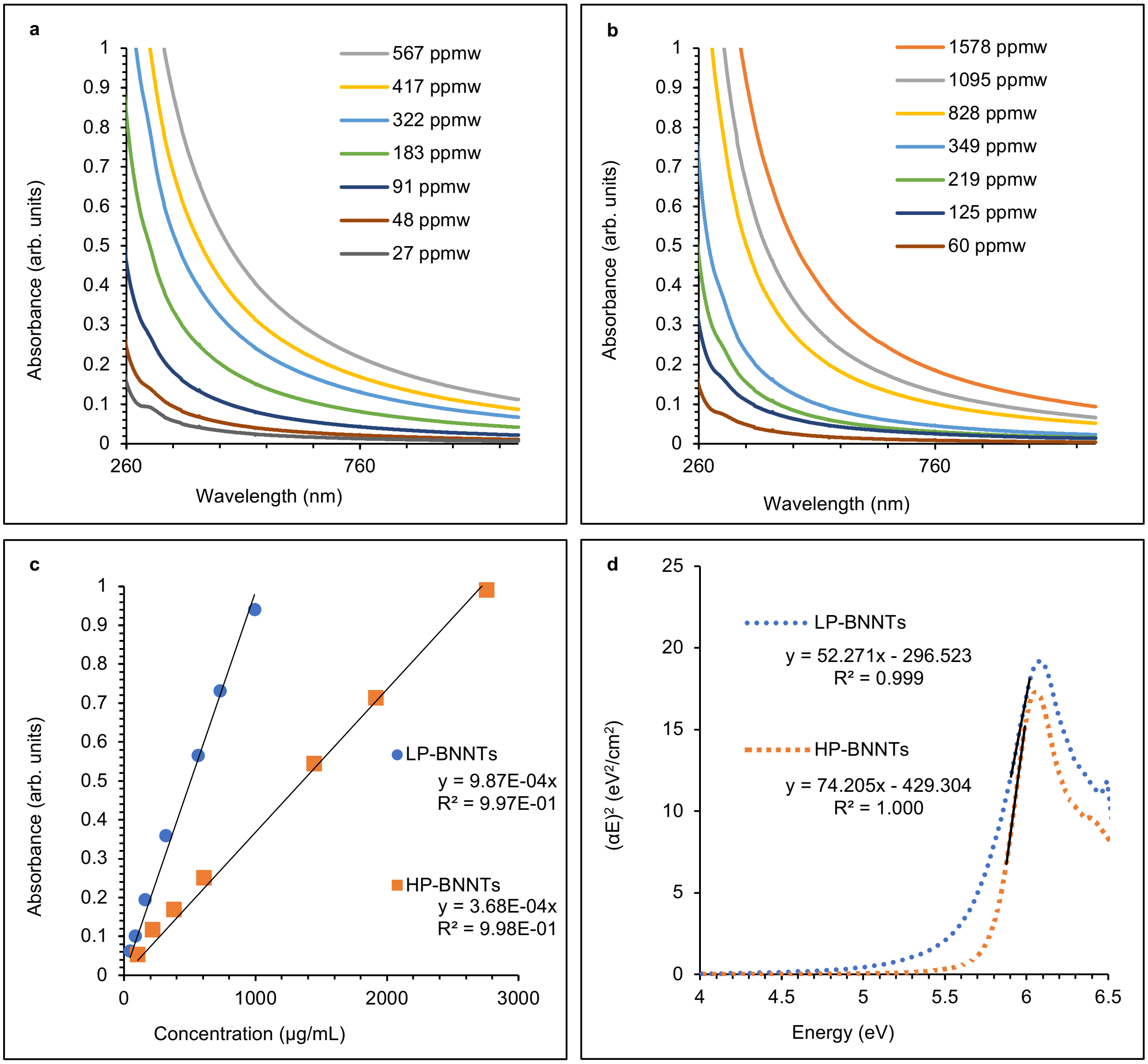


**Supplementary Fig. 3 UV-visible spectroscopy (UV-vis). a** UV-vis absorbance spectra of lightly purified BNNTs (LP-BNNTs) in chlorosulfonic acid (CSA) at various concentrations measured with a 1 mm path length. **b** UV-vis absorbance spectra of highly purified BNNTs (HP-BNNTs) in CSA at various concentrations measured with a 1 mm path length. The absorbance cutoff for CSA (A = 1 a.u.) was measured to be 259 nm using a 1 cm path length cuvettes with water in the reference cell. **c** Linear fits to the absorbances of LP-BNNTs and HP-BNNTs at 350 nm used determine extinction coefficients of $9.87\times10^{-3}$ mL·µg$^{-1}$·cm$^{-1}$ and 3.68 $\times10^{-3}$ mL·µg$^{-1}$·cm$^{-1}$, respectively. **d** Tauc plot of recovered (previously dissolved in CSA) LP-BNNTs and HP-BNNTs in 1 wt% aqueous SDS at 4 ppmw and 2 ppmw, respectively.

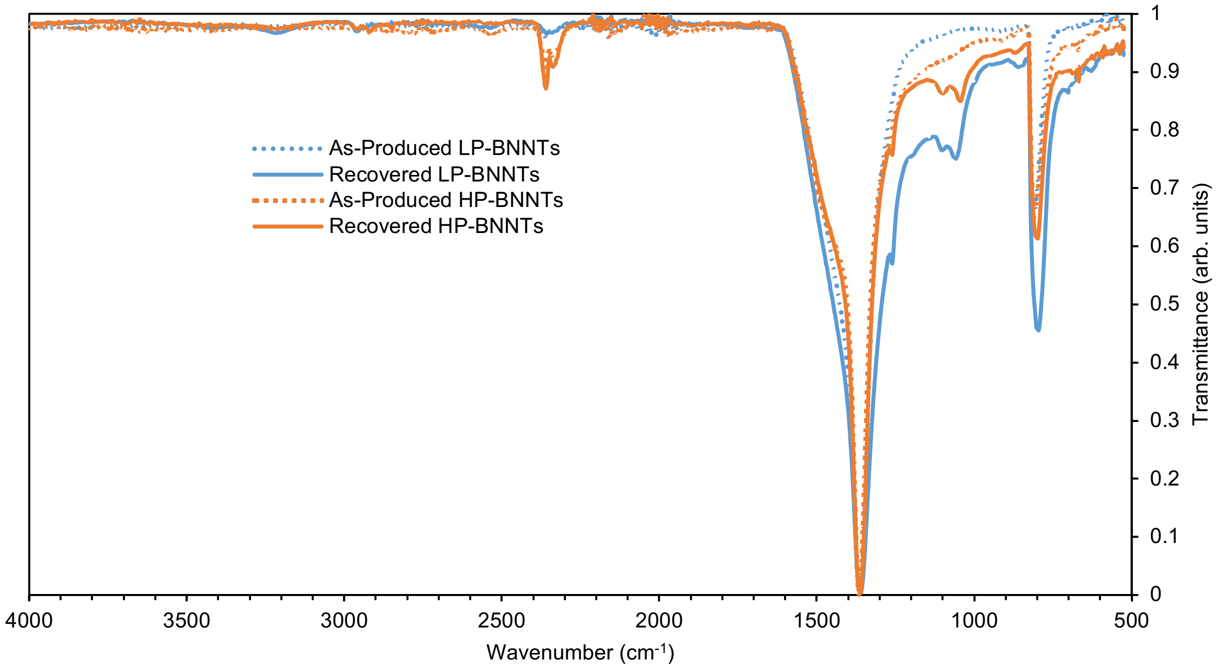


**Supplementary Fig. 4 Fourier transform infrared spectroscopy (FTIR).** FTIR spectra of as produced and recovered (previously exposed to CSA) BNNTs. Spectra are normalized to the intensity of the peak at 1350 $cm^{-1}$.

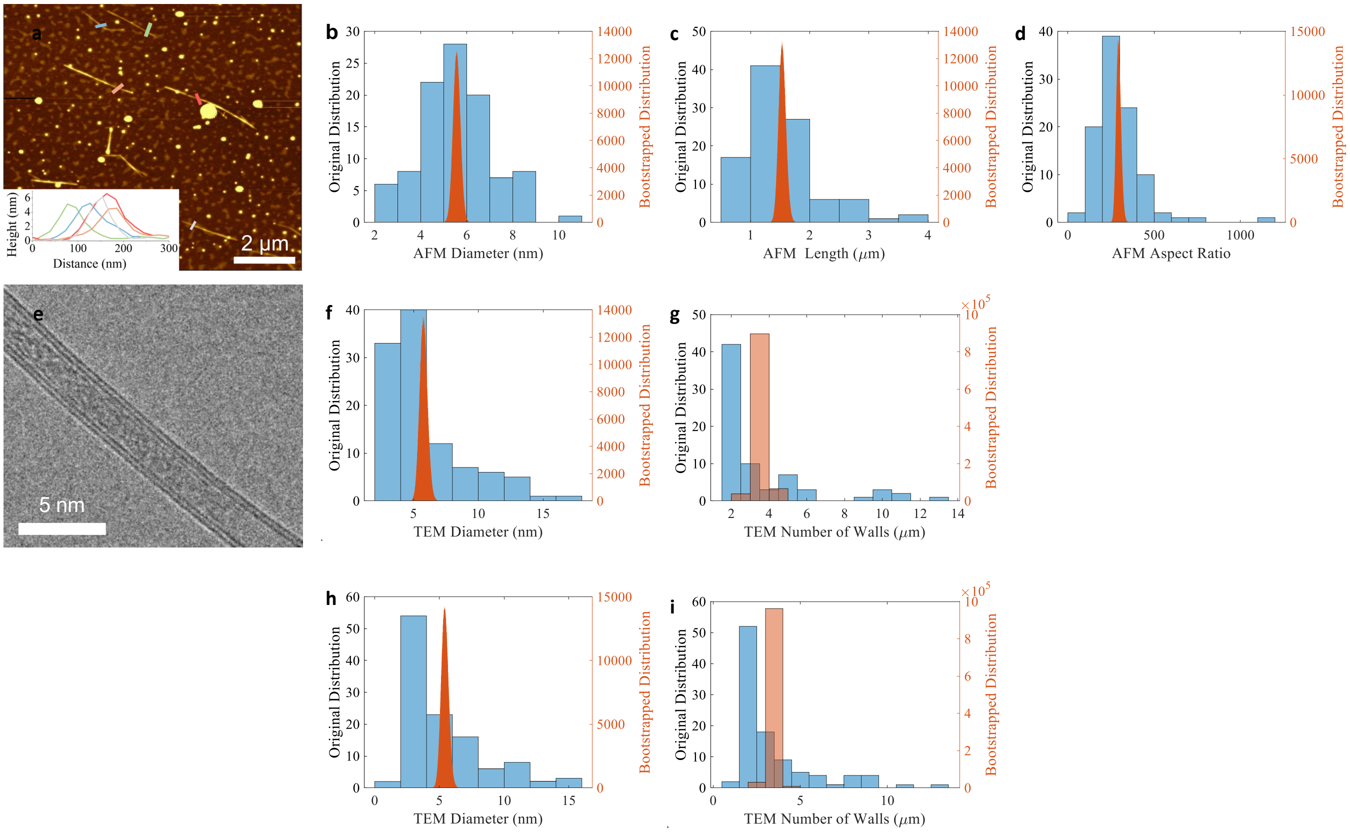


**Supplementary Fig. 5 Number of walls, length, diameter distributions.** Atomic force microscopy (AFM) and transmission electron microscopy (TEM) of BNNTs are used to determine BNNT properties for use in conjunction with capillary extensional rheology. **a** Typical AFM image on mica with inset line scan. **b** AFM diameter distribution for highly purified BNNTs (HP-BNNTs). **c** AFM length distribution for HP-BNNTs. **d** AFM aspect ratio distribution for HP-BNNTs. **E.** Typical TEM image for HP-BNNTs. **f** TEM diameter distribution for HP-BNNTs. **g** TEM number of walls distribution for HP-BNNTs. **h** TEM diameter distribution for lightly purified BNNTs (LP-BNNTs). **i** TEM number of wall distribution for LP-BNNTs. LP-BNNTs have slightly smaller average diameter (5.4 nm ± 0.3 nm) and number of walls (3.4 ± 0.2), which may be a result of purification etching the outer walls of multi-walled BNNTs. The average diameters for both HP-BNNT and LP-BNNT agree within measurement error.

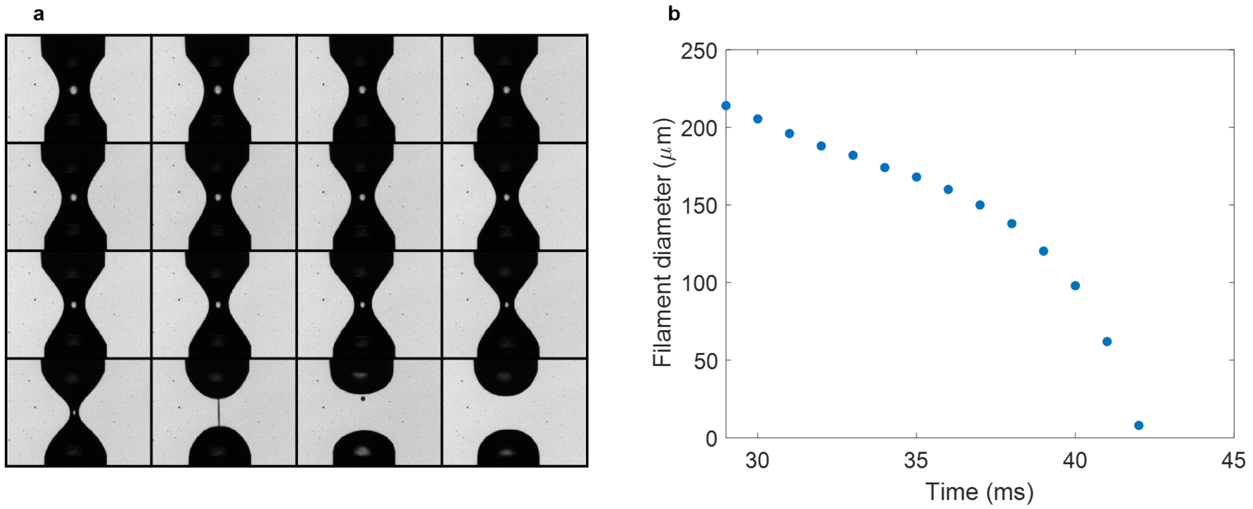


**Supplementary Fig. 6 Capillary thinning rheometry. a** Frames of a representative high speed video (1000 fps) during capillary thinning rheometry of a solution of highly purified BNNTs (HP-BNNTs) at 7260 ppmw in chlorosulfonic acid (CSA). **b** Plot of filament diameter. Sequence of frames is left to right, starting from the top row. The parameters for aspect ratio determination from capillary thinning rheology analysis are based off the following average property values from microscopic characterization: number of walls = 3.5; diameter = 5.8 nm; BNNT density = 1.50 $g/cm^3$.

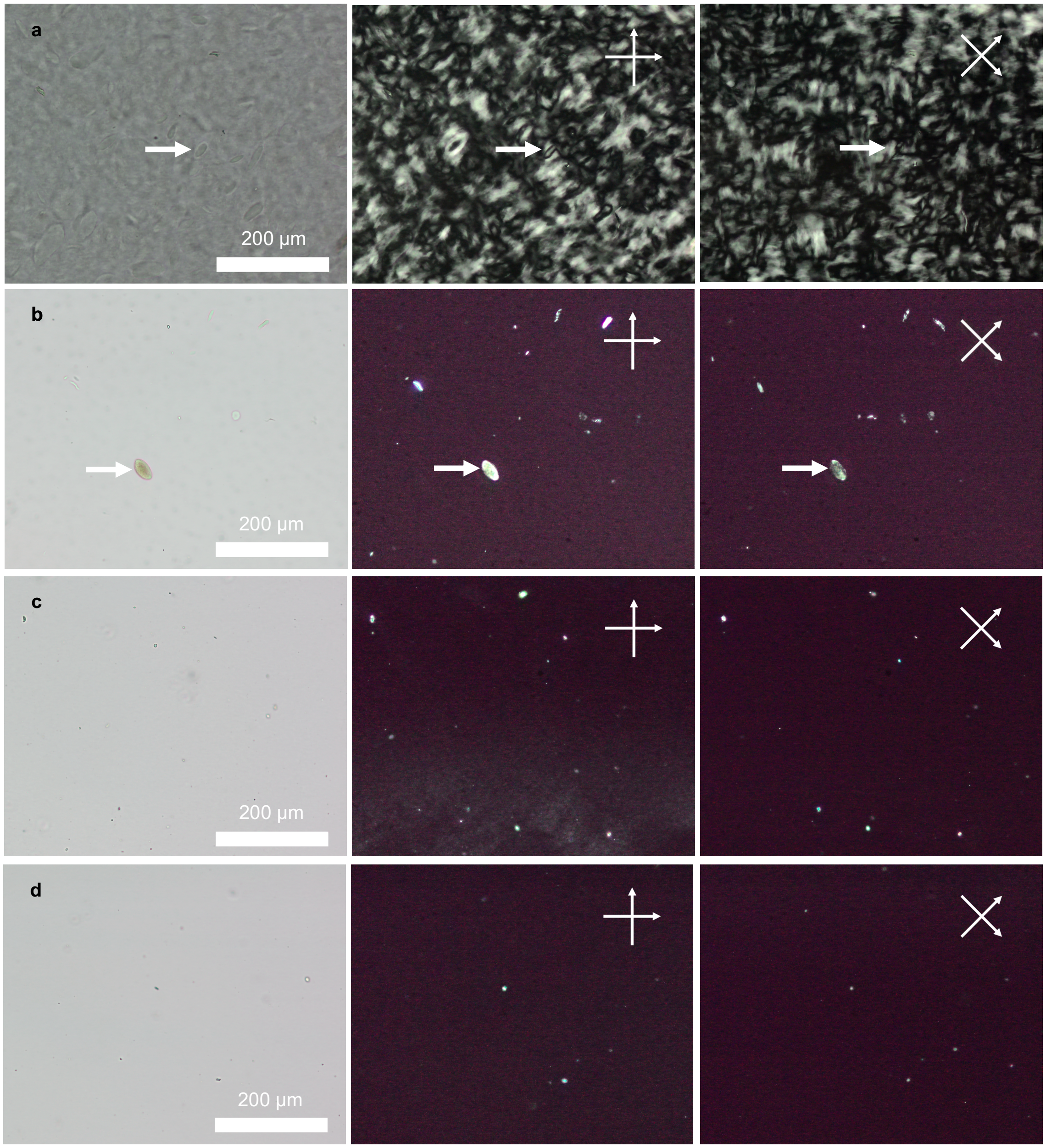


**Supplementary Fig. 7 Tactoid persistence and isotropic cloud point transition.** Polarized light microscopy (PLM) of highly purified BNNTs (HP-BNNTs) in chlorosulfonic acid (CSA) in flame-sealed 1 mm × 0.1 mm rectangular capillaries: unpolarized transmitted light and polarized light images with polarizer/analyzer at 0°/90° and 45°/135°, as depicted by the white crossed arrows. **a** Solution of HP-BNNTs in CSA at 7260 ppmw with no capillary sonication, showing the presence of tactoids (white arrows). **b** Solution of HP-BNNTs in CSA at 50 ppmw with no sonication, showing the presence of tactoids (white arrows). **c** Solution of HP-BNNTs in CSA at 250 ppmw showing faint birefringence in the lower half of the field of view. **d** Solution of HP-BNNTs in CSA at 90 ppmw showing no birefringence for the whole field of view. The isotropic cloud point is determined as the midpoint between these two concentrations, 170 ppmw (220 ppmv).

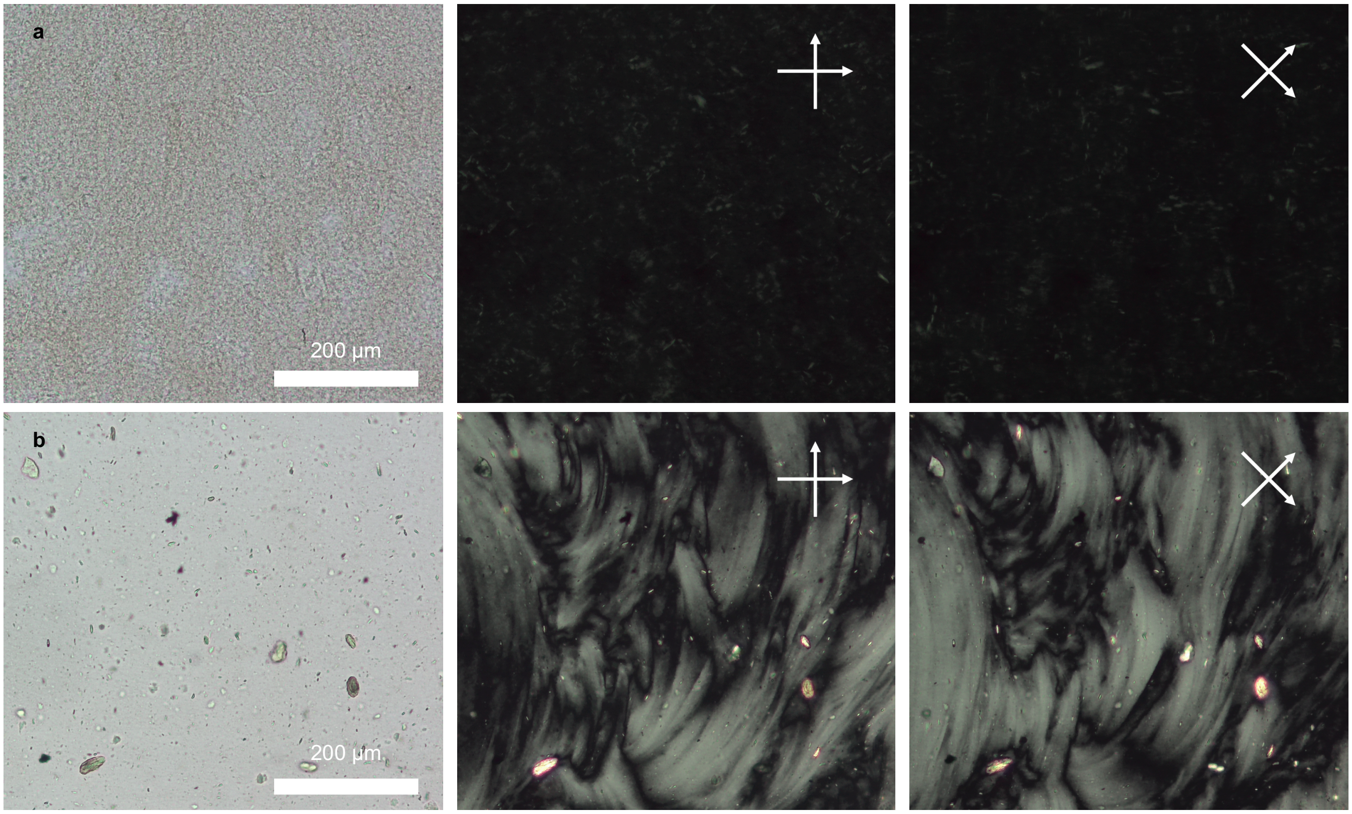


**Supplementary Fig. 8 Induced nematic alignment in lightly purified BNNT (LP-BNNT) solutions.** Polarized light microscopy (PLM) of lightly purified BNNTs (LP-BNNTs) in chlorosulfonic acid (CSA) (same as Fig. 2a)in flame-sealed 1 mm × 0.1 mm rectangular capillaries: unpolarized transmitted light and polarized light images with polarizer/analyzer at 0°/90° and 45°/135°, as depicted by the white crossed arrows. **a** 7000 ppmw solution of LP-BNNTs in chlorosulfonic acid (CSA) before capillary sonication appears isotropic because of low-intensity birefringence. **b** The same solution after capillary sonication showing birefringent nematic domains spanning the width of the capillary. Undissolved aggregates are present throughout the field of view.

## Supplementary References


1. Blase, X., Rubio, A., Louie, S. G. & Cohen, M. L. Stability and band gap constancy of boron nitride nanotubes. *Epl* **28**, 335–340 (1994).
2. Arenal, R. *et al.* Optical Gap Measurements on Individual Boron Nitride Nanotubes by Electron Energy Loss Spectroscopy. *Microsc. Microanal.* **14**, 274–282 (2008).
3. Nautiyal, P. *et al.* Oxidative Unzipping and Transformation of High Aspect Ratio Boron Nitride Nanotubes into "White Graphene Oxide" Platelets. *Sci. Rep.* **6**, 29498 (2016).
4. Efron, B. Bootstrap Methods: Another Look at the Jackknife. *Ann. Stat.* **7**, 1–26 (1979).
5. Bengio, E. A. *et al.* Statistical Length Measurement Method by Direct Imaging of Carbon Nanotubes. *ACS Appl. Mater. Interfaces* **6**, 6139–6146 (2014).
6. Tsentalovich, D. E. *et al.* Relationship of Extensional Viscosity and Liquid Crystalline Transition to Length Distribution in Carbon Nanotube Solutions. *Macromolecules* **49**, 681–689 (2016).
7. Davis, V. A. *et al.* True solutions of single-walled carbon nanotubes for assembly into macroscopic materials. *Nat. Nanotechnol.* **4**, 830–834 (2009).
